# Supplementary material for: Risk Factors for Problematic Drinking in One’s Thirties and Forties: A Longitudinal Analysis of the 1970 British Cohort Study
Source: Int J Environ Res Public Health. 2022 Aug 26;19(17):10664. doi: 10.3390/ijerph191710664 (PMC9518189; doi:10.3390/ijerph191710664)
Supplement: Supplementary file 1 [file ijerph-19-10664-s001.zip › ijerph-1854033-supplementary.pdf]

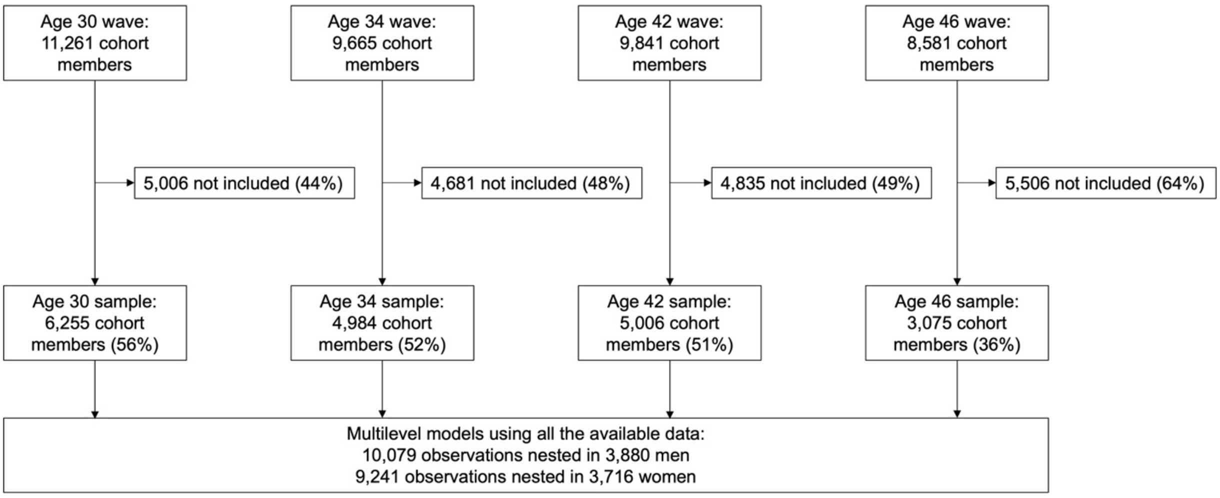

Supplemental Figure S1. Participant flow. Cohort members were not included in a given wave if they were missing data for the dependent variable and the independent variables.

Supplemental Table S1. Problematic drinking frequency in cohort members who were and were not included in the present analysis

Problematic drinking, No. (%)		Included	Not included
Age 30			
	No	5,382 (86.04)	4,315 (86.20)
	Yes	873 (13.96)	691 (13.80)
	Total	6,255 (100.00)	5,006 (100.00)
	Missing	0	0
Age 34			
	No	4,070 (81.66)	3,877 (82.82)
	Yes	914 (18.34)	804 (17.18)
	Total	4,984 (100.00)	4,681 (100.00)
	Missing	0	0
Age 42			
	No	3,764 (75.19)	2,858 (76.93)
	Yes	1,242 (24.81)	857 (23.07)
	Total	5,006 (100.00)	3,715 (100.00)
	Missing	0	1,120
Age 46			
	No	2,339 (76.07)	4,173 (76.95)
	Yes	736 (23.93)	1,250 (23.05)
	Total	3,075 (100.00)	5,423 (100.00)
	Missing	0	83

Supplemental Table S2. Longitudinal associations of modifiable and non-modifiable risk factors with problematic drinking in men: sensitivity analysis excluding cohort members with problematic drinking at age 30

Potential risk factor	Odds ratio (95% confidence interval)
Smoking	
Never smoked	Reference
Former smoker	2.71 (2.12, 3.47)
Current smoker	3.50 (2.70, 4.54)
Leisure time physical activity	
None	Reference
Once a week or less	1.07 (0.83, 1.48)
Two or three times a week	1.03 (0.79, 1.34)
Four or more times a week	0.92 (0.71, 1.18)
Highest academic qualification	
None	Reference
GCSE or equivalent	0.70 (0.53, 0.91)
A-level or equivalent	0.68 (0.47, 0.98)
Degree or higher degree	0.94 (0.68, 1.29)
Occupation	
Unskilled, semi-skilled or skilled	Reference
Managerial or professional	0.90 (0.73, 1.12)

Cohabiting as a couple		
	No	Reference
	Yes	0.89 (0.71, 1.11)
Malaise score		
	Low	Reference
	High	1.33 (1.00, 1.78)
Mother drank during pregnancy		
	No	Reference
	Yes	1.69 (1.36, 2.10)
Father's occupation in 1970		
	Unskilled, semi-skilled or skilled	Reference
	Managerial or professional	1.03 (0.81, 1.30)

Potential risk factors were assessed at age 30, age 34, age 42, and age 46. The cases of problematic drinking were as follows: 307 of 2,155 (14.25%) cohort members at age 34; 620 of 2,109 (29.40%) cohort members at age 42; and 343 of 1,261 (27.20%) cohort members at age 46. A multilevel model was fitted to the data, which was a linear model that allowed for random intercepts. All variables in the model were time varying except mother drank during pregnancy and father's occupation in 1970. The model included 8,228 observations in 3,191 male cohort members. The average number of observations per cohort member was 2.6, where the minimum was 1 and the maximum was 4. Values are mutually adjusted odds ratios. GCSE is general certificate of education, a qualification usually sought around 16 years of age. A-level is advance level, a qualification usually sought around 18 years of age.

Supplemental Table S3. Longitudinal associations of modifiable and non-modifiable risk factors with problematic drinking in women: sensitivity analysis excluding cohort members with problematic drinking at age 30

Potential risk factor		Odds ratio (95% confidence interval)
Smoking		
	Never smoked	Reference
	Former smoker	2.81 (2.15, 3.66)
	Current smoker	4.81 (3.62, 6.37)
Leisure time physical activity		
	None	Reference
	Once a week or less	1.01 (0.73, 1.39)
	Two or three times a week	1.13 (0.86, 1.48)
	Four or more times a week	1.23 (0.93, 1.61)
Highest academic qualification		
	None	Reference
	GCSE or equivalent	1.10 (0.81, 1.50)
	A-level or equivalent	0.77 (0.52, 1.14)
	Degree or higher degree	1.46 (1.02, 2.10)
Occupation		
	Unskilled, semi-skilled or skilled	Reference
	Managerial or professional	1.23 (0.98, 1.55)

Cohabiting as a couple		
	No	Reference
	Yes	0.86 (0.67, 1.09)
Malaise score		
	Low	Reference
	High	1.64 (1.26, 2.12)
Mother drank during pregnancy		
	No	Reference
	Yes	1.45 (1.15, 1.82)
Father's occupation in 1970		
	Unskilled, semi-skilled or skilled	Reference
	Managerial or professional	0.90 (0.70, 1.15)

Potential risk factors were assessed at age 30, age 34, age 42, and age 46. The cases of problematic drinking were as follows: 210 of 2,146 (9.79%) cohort members at age 34; 323 of 2,305 (14.01%) cohort members at age 42; and 201 of 1,400 (14.36%) cohort members at age 46. A multilevel model was fitted to the data, which was a linear model that allowed for random intercepts. All variables in the model were time varying except mother drank during pregnancy and father's occupation in 1970. The model included 8,530 observations in 3,436 female cohort members. The average number of observations per cohort member was 2.5, where the minimum was 1 and the maximum was 4. Values are mutually adjusted odds ratios. GCSE is general certificate of education, a qualification usually sought around 16 years of age. A-level is advance level, a qualification usually sought around 18 years of age.
